# *N*-(4,5-Diaza­fluoren-9-yl­idene)aniline

**DOI:** 10.1107/S1600536808016668

**Published:** 2008-06-07

**Authors:** Hui Cang, Dong Jin, Si-Qing Wang, Shan Liu, Jin-Tang Wang

**Affiliations:** a Department of Applied Chemistry, College of Science, Nanjing University of Technology, Nanjing 210009, People’s Republic of China; b College of Chemistry and Chemical Engineering, Nanjing University of Technology, Nanjing 210009, People’s Republic of China

## Abstract

In the mol­ecule of the title compound, C_17_H_11_N_3_, the 4,5-diaza­fluorenyl­idene unit is nearly planar and is oriented with respect to the phenyl ring at a dihedral angle of 75.75 (3)°. In the crystal structure, the mol­ecules are aligned in the [100] direction in such a way that neighbouring 4,5-diaza­fluorenyl­idene planes face each other in an anti­parallel fashion.

## Related literature

For related literature, see: Wang & Rillema (1997 ▶); Wang *et al.* (2006 ▶); Peters *et al.* (1998 ▶); Glagovich *et al.* (2004*a* ▶,*b* ▶). For bond-length data, see: Allen *et al.* (1987 ▶).
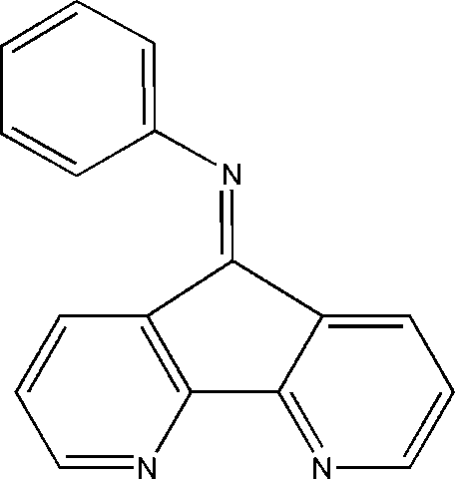


## Experimental

### 

#### Crystal data

C_17_H_11_N_3_
*M* *_r_* = 257.29
Triclinic, 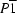

*a* = 7.1950 (14) Å
*b* = 8.5860 (17) Å
*c* = 11.876 (2) Å
α = 80.63 (3)°
β = 74.78 (3)°
γ = 66.46 (3)°
*V* = 647.6 (2) Å^3^
*Z* = 2
Mo *K*α radiation
μ = 0.08 mm^−1^
*T* = 298 (2) K
0.20 × 0.10 × 0.05 mm

#### Data collection

Enraf–Nonius CAD-4 diffractometer
Absorption correction: ψ scan (North *et al.*, 1968 ▶) *T*_min_ = 0.984, *T*_max_ = 0.996
2529 measured reflections
2326 independent reflections
1642 reflections with *I* > 2σ(*I*)
*R*_int_ = 0.057
3 standard reflections frequency: 120 min intensity decay: none

#### Refinement

*R*[*F* ^2^ > 2σ(*F* ^2^)] = 0.056
*wR*(*F* ^2^) = 0.178
*S* = 1.02
2326 reflections
181 parameters
H-atom parameters constrained
Δρ_max_ = 0.19 e Å^−3^
Δρ_min_ = −0.25 e Å^−3^

### 

Data collection: *CAD-4 Software* (Enraf–Nonius, 1989 ▶); cell refinement: *CAD-4 Software*; data reduction: *XCAD4* (Harms & Wocadlo, 1995 ▶); program(s) used to solve structure: *SHELXS97* (Sheldrick, 2008 ▶); program(s) used to refine structure: *SHELXL97* (Sheldrick, 2008 ▶); molecular graphics: *SHELXTL* (Sheldrick, 2008 ▶); software used to prepare material for publication: *SHELXTL*.

## Supplementary Material

Crystal structure: contains datablocks I, global. DOI: 10.1107/S1600536808016668/hk2468sup1.cif

Structure factors: contains datablocks I. DOI: 10.1107/S1600536808016668/hk2468Isup2.hkl

Additional supplementary materials:  crystallographic information; 3D view; checkCIF report

## Figures and Tables

**Fig. 1.**
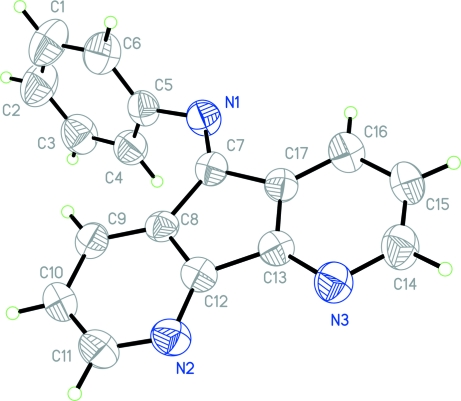
The molecular structure of the title molecule, with the atom-numbering scheme. Displacement ellipsoids are drawn at the 50% probability level.

**Fig. 2.**
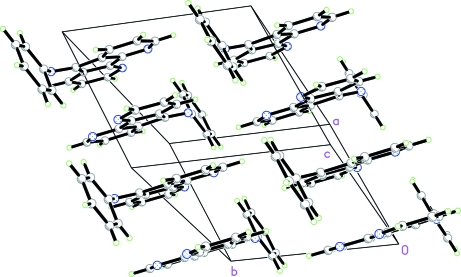
A packing diagram of (I).

## supplementary crystallographic information

### Comment

*N*-(4,5-diazafluorenylidene)benzenamine, is one of the important ligands, being utilized to synthesize complexes with interesting photochemical properties (Wang & Rillema, 1997). The crystal structure of 4-methyl-N-(4,5-diazafluorenylidene)benzenamine monohydrate, (II) (Wang *et al.*,  2006) was reported, previously. We report herein the crystal structure of the title compound, (I).

In the molecule of (I) (Fig. 1), the bond lengths (Allen *et al.*, 1987) and angles are within normal ranges, which are comparable with the corresponding values in other fluorenylidene compounds (II), N-fluorenylidene-aniline-benzene (4/1), (III) (Peters *et al.*,  1998), N-(9H-fluoren-9-ylidene)-N-(4-methoxyphenyl)amine, (IV) (Glagovich *et al.*,  2004*a*) and N-9H-fluoren-9-ylidene-3,4-dimethyl-aniline, (V) (Glagovich *et al.*, 2004*b*). Rings A (C1–C6), B (N2/C8–C12), C (C7/C8/C12/C13/C17) and D (N3/C13–C17) are, of course, planar. In the 4,5-diazafluorenylidene unit, the dihedral angles between the rings are B/C = 0.29 (3)°, C/D = 2.30 (3)° and B/D = 2.15 (3)°. So, rings B, C and D are nearly coplanar. The coplanar ring system is oriented with respect to ring A at a dihedral angle of 75.75 (3)°, in which it is reported as 65.1 (1)° in (II).

In the crystal structure, the molecules are aligned in the [100] direction, in such a way that neighbouring 4,5-diazafluorenylidene planes face in anti-parallel fashion (Fig. 2), as in (II).

### Experimental

The title compound, (I), was prepared according to the literature method (Wang & Rillema, 1997). Crystals suitable for X-ray analysis were obtained by dissolving (I) (2.0 g, 6.3 mmol) in acetate ester solution (50 ml, 1.0 mol/L) and evaporating the solvent slowly at room temperature for about 5 d.

### Refinement

H atoms were positioned geometrically, with C—H = 0.93 Å for aromatic H, and constrained to ride on their parent atoms with U_iso_(H) = 1.2U_eq_(C).

### Crystal data

**Table:**

C_17_H_11_N_3_	*Z* = 2
*M**_r_* = 257.29	*F*_000_ = 268
Triclinic, *P*1	*D*_x_ = 1.319 Mg m^−^^3^
Hall symbol: -P 1	Mo *K*α radiation λ = 0.71073 Å
*a* = 7.1950 (14) Å	Cell parameters from 25 reflections
*b* = 8.5860 (17) Å	θ = 9–13º
*c* = 11.876 (2) Å	µ = 0.08 mm^−^^1^
α = 80.63 (3)º	*T* = 298 (2) K
β = 74.78 (3)º	Needle, colourless
γ = 66.46 (3)º	0.20 × 0.10 × 0.05 mm
*V* = 647.6 (2) Å^3^	

### Data collection

**Table:**

Enraf–Nonius CAD-4 diffractometer	*R*_int_ = 0.057
Radiation source: fine-focus sealed tube	θ_max_ = 25.2º
Monochromator: graphite	θ_min_ = 1.8º
*T* = 298(2) K	*h* = −8→8
ω/2θ scans	*k* = −9→10
Absorption correction: ψ scan(North *et al.*, 1968)	*l* = 0→14
*T*_min_ = 0.984, *T*_max_ = 0.996	3 standard reflections
2529 measured reflections	every 120 min
2326 independent reflections	intensity decay: none
1642 reflections with *I* > 2σ(*I*)	

### Refinement

**Table:**

Refinement on *F*^2^	Secondary atom site location: difference Fourier map
Least-squares matrix: full	Hydrogen site location: inferred from neighbouring sites
*R*[*F*^2^ > 2σ(*F*^2^)] = 0.056	H-atom parameters constrained
*wR*(*F*^2^) = 0.178	*w* = 1/[σ^2^(*F*_o_^2^) + (0.08*P*)^2^ + 0.4*P*] where *P* = (*F*_o_^2^ + 2*F*_c_^2^)/3
*S* = 1.02	(Δ/σ)_max_ < 0.001
2326 reflections	Δρ_max_ = 0.19 e Å^−^^3^
181 parameters	Δρ_min_ = −0.25 e Å^−^^3^
Primary atom site location: structure-invariant direct methods	Extinction correction: none

### Special details

**Table:**

Geometry. All e.s.d.'s (except the e.s.d. in the dihedral angle between two l.s. planes) are estimated using the full covariance matrix. The cell e.s.d.'s are taken into account individually in the estimation of e.s.d.'s in distances, angles and torsion angles; correlations between e.s.d.'s in cell parameters are only used when they are defined by crystal symmetry. An approximate (isotropic) treatment of cell e.s.d.'s is used for estimating e.s.d.'s involving l.s. planes.
Refinement. Refinement of F^2^ against ALL reflections. The weighted R-factor wR and goodness of fit S are based on F^2^, conventional R-factors R are based on F, with F set to zero for negative F^2^. The threshold expression of F^2^ > 2sigma(F^2^) is used only for calculating R-factors(gt) etc. and is not relevant to the choice of reflections for refinement. R-factors based on F^2^ are statistically about twice as large as those based on F, and R- factors based on ALL data will be even larger.

### Fractional atomic coordinates and isotropic or equivalent isotropic displacement parameters (Å^2^)

**Table:**

	*x*	*y*	*z*	*U*_iso_*/*U*_eq_	
N1	0.1000 (4)	1.2848 (3)	0.6315 (2)	0.0491 (6)	
N2	0.3187 (4)	0.7234 (3)	0.4917 (2)	0.0492 (6)	
N3	0.3994 (4)	0.9717 (3)	0.2888 (2)	0.0503 (6)	
C1	0.0047 (6)	1.2899 (6)	0.9516 (3)	0.0808 (12)	
H1B	0.0717	1.2935	1.0078	0.097*	
C2	−0.1937 (6)	1.2891 (5)	0.9852 (3)	0.0778 (11)	
H2B	−0.2605	1.2921	1.0638	0.093*	
C3	−0.2916 (6)	1.2841 (5)	0.9021 (3)	0.0675 (9)	
H3B	−0.4257	1.2845	0.9249	0.081*	
C4	−0.1951 (5)	1.2783 (4)	0.7855 (3)	0.0561 (8)	
H4A	−0.2637	1.2753	0.7301	0.067*	
C5	0.0048 (5)	1.2772 (3)	0.7513 (2)	0.0470 (7)	
C6	0.1040 (5)	1.2853 (4)	0.8350 (3)	0.0616 (9)	
H6A	0.2369	1.2877	0.8124	0.074*	
C7	0.1686 (4)	1.1567 (3)	0.5708 (2)	0.0401 (6)	
C8	0.1856 (4)	0.9756 (3)	0.6012 (2)	0.0385 (6)	
C9	0.1347 (4)	0.8860 (4)	0.7046 (2)	0.0454 (7)	
H9A	0.0739	0.9384	0.7750	0.055*	
C10	0.1782 (5)	0.7148 (4)	0.6987 (3)	0.0501 (7)	
H10A	0.1470	0.6498	0.7662	0.060*	
C11	0.2673 (5)	0.6404 (4)	0.5935 (3)	0.0530 (8)	
H11A	0.2936	0.5253	0.5929	0.064*	
C12	0.2774 (4)	0.8874 (3)	0.4993 (2)	0.0409 (6)	
C13	0.3186 (4)	1.0075 (3)	0.4004 (2)	0.0405 (6)	
C14	0.4125 (5)	1.1058 (4)	0.2161 (3)	0.0574 (8)	
H14A	0.4674	1.0878	0.1371	0.069*	
C15	0.3502 (5)	1.2689 (4)	0.2504 (3)	0.0538 (8)	
H15A	0.3631	1.3562	0.1951	0.065*	
C16	0.2688 (4)	1.3018 (4)	0.3668 (3)	0.0502 (7)	
H16A	0.2265	1.4099	0.3924	0.060*	
C17	0.2534 (4)	1.1661 (3)	0.4432 (2)	0.0398 (6)	

### Atomic displacement parameters (Å^2^)

**Table:**

	*U*^11^	*U*^22^	*U*^33^	*U*^12^	*U*^13^	*U*^23^
N1	0.0548 (14)	0.0417 (13)	0.0454 (14)	−0.0158 (11)	−0.0029 (11)	−0.0071 (11)
N2	0.0500 (14)	0.0445 (14)	0.0527 (15)	−0.0180 (11)	−0.0079 (11)	−0.0066 (11)
N3	0.0466 (14)	0.0543 (15)	0.0451 (14)	−0.0190 (11)	0.0006 (11)	−0.0072 (11)
C1	0.079 (3)	0.109 (3)	0.048 (2)	−0.027 (2)	−0.0153 (19)	−0.010 (2)
C2	0.080 (3)	0.102 (3)	0.046 (2)	−0.032 (2)	−0.0057 (18)	−0.0088 (19)
C3	0.065 (2)	0.072 (2)	0.059 (2)	−0.0299 (18)	0.0026 (17)	−0.0032 (17)
C4	0.064 (2)	0.0534 (18)	0.0501 (18)	−0.0241 (15)	−0.0077 (15)	−0.0049 (14)
C5	0.0553 (17)	0.0351 (15)	0.0422 (16)	−0.0116 (12)	−0.0043 (13)	−0.0044 (12)
C6	0.0573 (19)	0.065 (2)	0.0533 (19)	−0.0150 (16)	−0.0067 (15)	−0.0076 (15)
C7	0.0352 (13)	0.0419 (15)	0.0407 (15)	−0.0114 (11)	−0.0091 (11)	−0.0023 (12)
C8	0.0351 (13)	0.0410 (14)	0.0432 (15)	−0.0152 (11)	−0.0133 (11)	−0.0029 (11)
C9	0.0472 (16)	0.0462 (16)	0.0405 (15)	−0.0140 (13)	−0.0111 (12)	−0.0034 (12)
C10	0.0534 (17)	0.0437 (16)	0.0510 (18)	−0.0192 (13)	−0.0116 (14)	0.0056 (13)
C11	0.0542 (18)	0.0402 (16)	0.061 (2)	−0.0160 (13)	−0.0102 (15)	−0.0026 (14)
C12	0.0334 (13)	0.0409 (15)	0.0465 (16)	−0.0125 (11)	−0.0073 (12)	−0.0038 (12)
C13	0.0311 (13)	0.0457 (16)	0.0429 (16)	−0.0142 (11)	−0.0040 (11)	−0.0054 (12)
C14	0.0544 (18)	0.067 (2)	0.0446 (17)	−0.0258 (16)	0.0040 (14)	−0.0045 (15)
C15	0.0518 (17)	0.0566 (19)	0.0495 (18)	−0.0250 (14)	−0.0032 (14)	0.0057 (14)
C16	0.0450 (16)	0.0456 (17)	0.0511 (18)	−0.0124 (13)	−0.0039 (13)	−0.0020 (13)
C17	0.0350 (13)	0.0431 (15)	0.0411 (15)	−0.0152 (11)	−0.0073 (11)	−0.0021 (11)

### Geometric parameters (Å, °)

**Table:**

N1—C7	1.269 (3)	C7—C17	1.480 (4)
N1—C5	1.410 (3)	C7—C8	1.500 (4)
N2—C12	1.331 (3)	C8—C9	1.384 (4)
N2—C11	1.343 (4)	C8—C12	1.398 (4)
N3—C13	1.333 (3)	C9—C10	1.385 (4)
N3—C14	1.341 (4)	C9—H9A	0.9300
C1—C2	1.380 (5)	C10—C11	1.375 (4)
C1—C6	1.381 (5)	C10—H10A	0.9300
C1—H1B	0.9300	C11—H11A	0.9300
C2—C3	1.369 (5)	C12—C13	1.482 (4)
C2—H2B	0.9300	C13—C17	1.386 (4)
C3—C4	1.375 (4)	C14—C15	1.385 (4)
C3—H3B	0.9300	C14—H14A	0.9300
C4—C5	1.385 (4)	C15—C16	1.380 (4)
C4—H4A	0.9300	C15—H15A	0.9300
C5—C6	1.392 (4)	C16—C17	1.379 (4)
C6—H6A	0.9300	C16—H16A	0.9300
			
C7—N1—C5	121.5 (2)	C8—C9—C10	117.0 (3)
C12—N2—C11	114.6 (2)	C8—C9—H9A	121.5
C13—N3—C14	114.1 (3)	C10—C9—H9A	121.5
C2—C1—C6	120.2 (4)	C11—C10—C9	120.4 (3)
C2—C1—H1B	119.9	C11—C10—H10A	119.8
C6—C1—H1B	119.9	C9—C10—H10A	119.8
C3—C2—C1	119.6 (3)	N2—C11—C10	124.1 (3)
C3—C2—H2B	120.2	N2—C11—H11A	117.9
C1—C2—H2B	120.2	C10—C11—H11A	117.9
C2—C3—C4	121.2 (3)	N2—C12—C8	125.8 (3)
C2—C3—H3B	119.4	N2—C12—C13	125.6 (2)
C4—C3—H3B	119.4	C8—C12—C13	108.6 (2)
C3—C4—C5	119.6 (3)	N3—C13—C17	125.4 (3)
C3—C4—H4A	120.2	N3—C13—C12	126.3 (2)
C5—C4—H4A	120.2	C17—C13—C12	108.3 (2)
C4—C5—C6	119.6 (3)	N3—C14—C15	124.7 (3)
C4—C5—N1	120.3 (3)	N3—C14—H14A	117.6
C6—C5—N1	119.8 (3)	C15—C14—H14A	117.6
C1—C6—C5	119.9 (3)	C16—C15—C14	119.8 (3)
C1—C6—H6A	120.1	C16—C15—H15A	120.1
C5—C6—H6A	120.1	C14—C15—H15A	120.1
N1—C7—C17	122.3 (2)	C17—C16—C15	116.6 (3)
N1—C7—C8	132.7 (2)	C17—C16—H16A	121.7
C17—C7—C8	105.0 (2)	C15—C16—H16A	121.7
C9—C8—C12	118.0 (2)	C16—C17—C13	119.3 (3)
C9—C8—C7	133.7 (2)	C16—C17—C7	130.9 (3)
C12—C8—C7	108.3 (2)	C13—C17—C7	109.7 (2)
			
C6—C1—C2—C3	−0.1 (6)	C9—C8—C12—N2	0.9 (4)
C1—C2—C3—C4	0.5 (6)	C7—C8—C12—N2	179.7 (2)
C2—C3—C4—C5	0.2 (5)	C9—C8—C12—C13	−179.9 (2)
C3—C4—C5—C6	−1.3 (5)	C7—C8—C12—C13	−1.1 (3)
C3—C4—C5—N1	−175.0 (3)	C14—N3—C13—C17	−0.7 (4)
C7—N1—C5—C4	−75.0 (4)	C14—N3—C13—C12	177.4 (3)
C7—N1—C5—C6	111.3 (3)	N2—C12—C13—N3	1.2 (4)
C2—C1—C6—C5	−1.0 (6)	C8—C12—C13—N3	−177.9 (2)
C4—C5—C6—C1	1.7 (5)	N2—C12—C13—C17	179.7 (2)
N1—C5—C6—C1	175.5 (3)	C8—C12—C13—C17	0.5 (3)
C5—N1—C7—C17	174.9 (2)	C13—N3—C14—C15	0.2 (4)
C5—N1—C7—C8	−4.7 (5)	N3—C14—C15—C16	0.4 (5)
N1—C7—C8—C9	−0.6 (5)	C14—C15—C16—C17	−0.4 (4)
C17—C7—C8—C9	179.8 (3)	C15—C16—C17—C13	0.0 (4)
N1—C7—C8—C12	−179.1 (3)	C15—C16—C17—C7	−177.7 (3)
C17—C7—C8—C12	1.2 (3)	N3—C13—C17—C16	0.7 (4)
C12—C8—C9—C10	−0.3 (4)	C12—C13—C17—C16	−177.8 (2)
C7—C8—C9—C10	−178.7 (3)	N3—C13—C17—C7	178.8 (2)
C8—C9—C10—C11	−0.3 (4)	C12—C13—C17—C7	0.3 (3)
C12—N2—C11—C10	0.3 (4)	N1—C7—C17—C16	−2.9 (4)
C9—C10—C11—N2	0.3 (5)	C8—C7—C17—C16	176.9 (3)
C11—N2—C12—C8	−0.9 (4)	N1—C7—C17—C13	179.3 (2)
C11—N2—C12—C13	−180.0 (2)	C8—C7—C17—C13	−1.0 (3)
